# Age-related terminal duct lobular unit involution in benign tissues from Chinese breast cancer patients with luminal and triple-negative tumors

**DOI:** 10.1186/s13058-017-0850-5

**Published:** 2017-05-25

**Authors:** Changyuan Guo, Hyuna Sung, Shan Zheng, Jennifer Guida, Erni Li, Jing Li, Nan Hu, Joseph Deng, Jonine D. Figueroa, Mark E. Sherman, Gretchen L. Gierach, Ning Lu, Xiaohong R. Yang

**Affiliations:** 1 National Cancer Center/Cancer Hospital, Chinese Academy of Medical Sciences and Peking Union Medical College, Beijing, China; 2 Division of Cancer Epidemiology & Genetics, National Cancer Institute, National Institutes of Health, Bethesda, MD USA; 3 Usher Institute of Population Health Sciences and Informatics, The University of Edinburgh, Edinburgh, UK; 4 Health Sciences Research, Mayo Clinic, Jacksonville, FL USA

**Keywords:** Terminal ductal lobular unit (TDLU) involution, Breast cancer, Tumor subtype, Luminal tumor, Triple-negative tumor, Core basal phenotype

## Abstract

**Background:**

Terminal duct lobular unit (TDLU) involution is a physiological process of breast tissue aging characterized by a reduction in the epithelial component. In studies of women with benign breast disease, researchers have found that age-matched women with lower levels of TDLU involution are at increased risk of developing breast cancer. We previously showed that breast cancer cases with core basal phenotype (CBP; estrogen receptor negative [ER^−^], progesterone receptor-negative [PR^−^], human epidermal growth factor receptor 2-negative [HER2^−^], cytokeratins (CK 5 or CK5/6)-positive [CK5/6^+^] and/or epidermal growth factor receptor-positive [EGFR^+^]) tumors had significantly reduced TDLU involution compared with cases with luminal A (ER^+^ and/or PR^+^, HER2^−^, CK5/6^−^, EGFR^−^) tumors from a population-based case-control study in Poland. We evaluated the association of TDLU involution with tumor subtypes in an independent population of women in China, where the breast cancer incidence rate, prevalence of known risk factors, and mammographic breast density are thought to be markedly different from those of Polish women.

**Methods:**

We performed morphometric assessment of TDLUs by using three reproducible semiquantitative measures that inversely correlate with TDLU involution (TDLU count/100 mm^2^, TDLU span in micrometer, and acini count/TDLU) by examining benign tissue blocks from 254 age-matched luminal A and 250 triple-negative (TN; ER^−^, PR^−^, HER2^−^, including 125 CBP) breast cancer cases treated in a tertiary hospital in Beijing, China.

**Results:**

Overall, we found that TN and particularly CBP cases tended to have greater TDLU measures (less involution) than luminal A cases in logistic regression models accounting for age, body mass index, parity, and tumor grade. The strongest association was observed for tertiles of acini count among younger women (aged <50 years) (CBP vs. luminal A; OR_trend_ 2.11, 95% CI 1.22–3.67, *P* = 0.008).

**Conclusions:**

These data extend previous findings that TN/CBP breast cancers are associated with reduced TDLU involution in surrounding breast parenchyma compared with luminal A cases among Chinese women, providing further support for differences in the pathogenesis of these tumor subtypes.

**Electronic supplementary material:**

The online version of this article (doi:10.1186/s13058-017-0850-5) contains supplementary material, which is available to authorized users.

## Background

Age-related terminal duct lobular unit (TDLU) involution is a physiological process of breast tissue aging by which the epithelial components of TDLUs atrophy and disappear. Importantly, TDLUs also represent the primary anatomical sources of breast cancers and their precursors [1]. In studies of women with benign breast disease, women with lesser degrees of TDLU involution had increased risk of subsequent breast cancer [2–4], and incorporation of involution in risk prediction has resulted in improved risk prediction [5]. Further, because individual variations in TDLU involution are also associated with breast cancer risk factors including age, menopausal status, parity, menopausal hormone therapy, and mammographic breast density (MD) [6–8], TDLU involution may represent an important marker of risk and an intermediate endpoint of breast cancer.

Breast cancer can be classified into different subtypes based on immunohistochemical expression of estrogen receptor (ER), progesterone receptor (PR), human epidermal growth factor receptor 2 (HER2), cytokeratins (CK5 or CK5/6), and epidermal growth factor receptor (EGFR). Among these subtypes, triple-negative (TN; ER^−^, PR^−^, HER2^−^) subtypes have been associated with features that contrast with those of luminal ER^+^ breast cancer (younger age at onset, higher prevalence of *BRAC1* germline mutations, and African American race) [9], distinct risk factors (greater and earlier parity, lack of breastfeeding, higher prevalence of positive family history of breast cancer) [10, 11], and worse prognosis [12]. Particularly, the core basal phenotype (CBP; ER^−^, PR^−^, HER2^−^, CK5/6^+^, and/or EGFR^+^) has been considered as displaying the most distinctive features compared with the luminal A subtype [12, 13].

In a previous study of Polish patients with breast cancer younger than age 55 years (232 luminal A and 49 CBP), we showed that TDLUs associated with CBP tumors had significantly reduced TDLU involution compared with parenchyma associated with luminal A tumors (ER^+^ and/or PR^+^, HER2^−^) [14]. Specifically, compared with luminal A tumors, CBP tumors tended to have a greater average number of acini per TDLU and a larger average TDLU span. The goal of the present study was to test the hypothesis that TDLU involution is reduced in breast parenchyma associated with CBP compared with luminal A tumors among Asian women, a group with lower reported incidence rates of breast cancer and generally smaller, more mammographically dense breasts than Western women [15].

## Methods

### Study population

Data and biologic specimens for the present analysis were collected from female patients with invasive breast cancer who had surgeries at Cancer Hospital, Chinese Academy of Medical Sciences (CHCAMS), Beijing, China, between 2009 and 2012. Eligible subjects included those who had a confirmed breast cancer diagnosis, had complete pathologic data, and had not received neoadjuvant therapies prior to surgeries. Breast cancer risk factors were extracted from medical records, which included age at diagnosis, body mass index (BMI), age at menarche, number of children, breastfeeding (yes or no), and first-degree family history of breast cancer. Pathologic data were retrieved from pathology reports, including tumor size; tumor grade; axillary lymph node status; and marker expression levels of ER, PR, HER2, CK5 (or CK 5/6), and EGFR. The status of ER, PR, CK5 (or CK 5/6), and EGFR expression in tumors was determined by immunohistochemistry (IHC), and >1% staining was considered as positive. HER2 expression was determined by IHC and fluorescence in situ hybridization (FISH), and either an IHC score of 3+ or a FISH-positive test result was defined as HER2^+^. To avoid misclassification, we excluded all HER2^+^ cases without FISH data. Basal marker (CK5 (or CK 5/6) and EGFR) information was available for women diagnosed between 2011 and 2012, and we identified all CBP cases (ER^−^, PR^−^, HER2^−^, CK5/6^+^, and/or EGFR^+^) in these two years. To increase power, we also identified TN cases defined by ER, PR, and HER2 (ER^−^, PR^−^, HER2^−^) from earlier years, resulting in a study set including a total of 328 TN/CBP cases. Given the aim of comparing TDLU involution associated with TN/CBP and luminal A breast cancers, we restricted the analysis to these two subtypes. We then performed 10-year age frequency matching to select luminal A cases (*n* = 328) that were defined as ER^+^ and/or PR^+^, HER2^−^, and negative for both basal markers. Tissue sections prepared from grossly benign breast tissue were stained with hematoxylin and eosin (H&E) and scanned to create digital image files. Cases with low-quality images (*n* = 11) or benign changes throughout the section (duct dilation, metaplasia, hyperplasia; *n* = 26), microscopic evidence of ductal carcinoma in situ (*n* = 32), or invasive cancer (*n* = 97) were excluded, resulting in 504 cases in the final analysis (254 luminal A and 250 TN/CBP cases [125 CBP]). The project was approved by the CHCAMS Ethics Committee and exempted from review by the Office for Human Research Protections at the National Institutes of Health because it did not involve interaction with human subjects and/or use of subjects’ personal identifying information (exempt number 11751).

### TDLU assessment

Grossly normal breast tissue blocks were retrieved, and H&E-stained tissue sections were scanned (Aperio ScanScope; Aperio Technologies, Vista, CA, USA) and loaded into Digital Image Hub software for review (SlidePath/Leica Microsystems, Dublin, Ireland). The lasso tool in Digital Image Hub was used to manually outline and measure total tissue area in square millimeters per section. TDLU involution was assessed using three semiquantitative measures with previously demonstrated reproducibility [4, 6]: TDLU count per unit area (count/100 mm^2^), TDLU span (measured with an electronic ruler in micrometers), and acini count per TDLU, all showing inverse correlations with TDLU involution [6]. Among women with observable normal TDLUs, up to ten sequential TDLUs were evaluated for acini count per TDLU (categories: 1 = 2–10, 2 = 11–20, 3 = 21–30, 4 = 31–50, 5 = 51–100, and 6 = >100) and TDLU span (maximum diameter of a TDLU), as previously described [6]. Both maximal and median values of acini count/TDLU and TDLU span were used as summary measures for each patient. All morphologic measurements were performed by a single trained cytotechnologist (R. Cora). To assess intrawoman concordance in TDLU involution measures, we evaluated two different benign sections within a single case that were available for 100 cases. Within-woman Spearman’s correlation coefficients (*r* ^2^) were 0.58 for TDLU count, 0.65 for median acini count/TDLU, and 0.69 for median TDLU span.

### Statistical analysis

The associations between breast cancer risk factors and TDLU involution measures were assessed using ordinal logistic regression models with tertiles of each TDLU involution measure as the outcome variable and breast cancer risk factors and clinical characteristics as explanatory variables. The associations between breast tumor subtype and TDLU involution measures were assessed using logistic regression with tumor subtype as the outcome variable (TN/CBP or CBP vs. luminal A) and TDLU involution measures as explanatory variables (comparing the second and third tertiles with the first tertile).

Trend *P* values, ORs, and 95% CIs were estimated, treating tertiles of TDLU involution measures as ordinal variables. Adjustment variables included potential confounders and/or tumor characteristics that were significantly associated with both TDLU measures and tumor subtypes (Table 1 and Additional file 1: Table S1): age (5-year intervals), BMI (<23, 23–24.9, 25–29.9, or 30+ kg/m^2^), parity (nulliparous vs. parous), and tumor grade (well, moderately, or poorly differentiated). To evaluate potential effect modification by age, we also performed stratified analysis by age (<50 vs. 50+ years), with cutpoints for tertiles determined based on distributions in each age group. All analyses were performed using SAS version 9.3 software (SAS Institute Inc., Cary, NC, USA).

**Table 1:** Distributions in measures of terminal duct lobular unit involution by breast cancer risk factors and tumor characteristics among Chinese patients with breast cancer

	Overall (*n* = 504)		Measures of TDLU involution							
			Count/100 mm^2^		Maximum span (μm)		Maximum category of acini count/TDLU^a^			
	Subjects (*n*)	%	Median	(IQR)	Median	(IQR)	1–3		4–6	
							Number	%	Number	%
Age										
All, mean (SD)	50.4	9.4								
20–39 years	48	9.5	30.8	(15.7–48.0)	924	(759.5–1308.5)	14	5.0	34	15.3
40–49 years	204	40.5	29.9	(17.4–44.6)	934.5	(712–1154)	71	25.2	133	59.9
50–59 years	176	34.9	12.3	(7.0–22.9)	674	(532.5–847.5)	131	46.5	45	20.3
60–79 years	76	15.1	8.7	(3.7–15.2)	559	(391.5–740)	66	23.4	10	4.5
*P* value^b^			<0.0001		<0.0001		<0.0001			
Body mass index										
All, mean (SD)	25.1	3.7								
< 23 kg/m^2^	155	30.9	19.2	(8.7–32.6)	773	(599–1075)	84	30.1	71	32.0
23–24.9 kg/m^2^	121	24.2	19.0	(10.2–37.2)	747	(565–964)	67	24.0	54	24.3
25–29.9 kg/m^2^	180	35.9	18.8	(9.1–36.8)	813.5	(613–1025)	98	35.1	82	36.9
30+ kg/m^2^	45	9.0	14.3	(8.3–27.2)	784	(566–946)	30	10.8	15	6.8
*P* value^b^			0.73		0.50		0.92			
Family history of breast cancer										
No	470	95.0	18.3	(8.7–34.8)	775	(591–1023)	265	95.0	205	94.9
Yes	25	5.1	17.1	(10.7–30.0)	751	(461–979)	14	5.0	11	5.1
*P* value^b^			0.76		0.12		0.55			
Age at menarche										
< 12 years	122	25.4	18.9	(10.8–38.6)	789.5	(603–1023)	68	24.7	54	26.2
12–13 years	215	44.7	19.7	(8.5–36.3)	796	(611–1080)	112	40.7	103	50.0
14+ years	144	29.9	15.4	(7.2–28.6)	713	(536.5–954)	95	34.6	49	23.8
*P* value^b^			0.99		0.99		0.94			
Parity/number of children										
Nulliparous	18	3.9	10.9	(4.6–36.6)	818	(482–1155)	11	4.2	7	3.4
One child	290	62.1	20.0	(11.1–36.3)	804.5	(612–1075)	147	56.3	143	69.4
More than one child	159	34.1	14.7	(7.3–33.8)	754	(547–955)	103	39.5	56	27.2
*P* value^b^			0.02		0.14		0.06			
Breastfeeding^c^										
No	19	6.2	15.4	(7.8–28.1)	565	(468–887)	14	8.1	5	3.8
Yes	286	93.8	18.3	(9.0–34.2)	790.5	(621–1025)	158	91.9	128	96.2
*P* value^b^			0.48		0.05		0.08			
Tumor subtype										
Luminal A	254	50.4	17.4	(8.3–32.8)	764.5	(600–992)	152	53.9	102	46.0
Triple-negative/core basal phenotype	250	49.6	19.5	(10.0–37.8)	803	(588–1043)	130	46.1	120	54.1
*P* value^b^			0.45		0.10		0.03			
Tumor size										
≤ 2 cm	277	56.4	18.3	(8.3–34.2)	777	(583–998)	160	58.4	117	53.9
> 2 cm	214	43.6	20.0	(10.1–35.8)	794	(606–1034)	114	41.6	100	46.1
*P* value^b^			0.71		0.76		0.19			
Lymph node invasion										
Negative	286	58.7	17.6	(9.4–34.9)	780.5	(566–989)	160	58.6	126	58.9
Positive	201	41.3	19.8	(8.4–34.1)	781	(604–1075)	113	41.4	88	41.1
*P* value^b^			0.86		0.27		0.81			
Grade										
Well differentiated	47	10.2	20.3	(8.3–40.0)	887	(644–1198)	26	10.0	21	10.5
Moderately differentiated	201	43.5	17.5	(9.4–36.0)	770	(599–979)	113	43.3	88	43.8
Poorly differentiated	214	46.3	18.7	(9.2–33.0)	774.5	(566–1025)	122	46.7	92	45.8
*P* value^b^			0.32		0.01		0.07			

*TDLU* Terminal duct lobular unit

^a^Categories for acini count/TDLU: 1 = 2–10, 2 = 11–20, 3 = 21–30, 4 = 31–50, 5 = 51–100, and 6 = >100

^b^ *P* values were obtained from ordinal logistic regression models using tertiles of TDLU involution measures as the outcome variable and age (5-year intervals), body mass index (<23, 23–24.9, 25–29.9, or 30+ kg/m^2^), parity (nulliparous, one child, and more than one child), tumor subtype (luminal A and triple-negative/core basal phenotype), and grade (well, moderately, or poorly differentiated) as explanatory variables

^c^Parous women only

## Results

In this study population including 504 breast cancer cases (254 luminal A and 250 TN/CBP), the mean (SD) age at diagnosis was 50.4 (9.4) years, and mean (SD) BMI was 25.1 (3.7) kg/m^2^. The majority of women did not report a family history of breast cancer (95%), had one (62.1%) or more than one (34.1%) child, and had breastfed their children (93.8%). Table 1 shows the distribution of TDLU involution measures by breast cancer risk factors and tumor characteristics. In the multivariable model including all examined risk and clinical factors, all three TDLU involution measures were inversely associated with age. Parity, breastfeeding, tumor subtype, and tumor grade were associated with some TDLU involution measures. Specifically, subjects who were parous (particularly having one child) were more likely to have a greater number of TDLUs and acini per TDLU than nulliparous women. Among parous women, women who had breastfed had larger TDLUs and a greater number of acini per TDLU than women who had never breastfed. Subjects with TN/CBP or higher-grade tumors also tended to have larger TDLUs and a greater number of acini per TDLU.

Table 2 shows the association between tumor subtype and measures of TDLU involution among all cases after accounting for potential confounders such as age, parity, BMI, and tumor grade. TN/CBP cases, in particular CBP cases, were more likely to have greater TDLU count, larger TDLU span, and greater number of acini per TDLU (all related to less involution) than luminal A cases, although most comparisons were not statistically significant. The strongest association was observed for maximum number of acini per TDLU (OR_trend_ 1.42, 95% CI 1.01–2.01, *P*_trend_ 0.045 for TN/CBP; OR_trend_ 1.69, 95% CI 1.12–2.55, *P*_trend_ 0.012 for CBP vs. luminal A cases). When analyzing younger and older women separately (Table 3), we found that the association of reduced TDLU involution and CBP was observed only among women younger than age 50 years, with the strongest effect found for maximum category of acini per TDLU when we compared CBP and luminal A cases (OR_T3 vs. T1_ 4.39, 95% CI 1.45–13.23, *P* = 0.009; OR_trend_ 2.11, 95% CI 1.22–3.67, *P*_trend_ = 0.008). Among older women, statistically significant associations between measures of TDLU involution and tumor subtype were not apparent. Similar but slightly weakened associations were observed when we used median values as summary measures for TDLU span and acini count/TDLU (Additional file 1: Table S2).

**Table 2:** Association of terminal duct lobular unit involution with breast tumor subtypes among overall cases

	Overall (*n* = 504)		Luminal A (*n* = 254)		TN/CBP (*n* = 250)		TN/CBP vs. luminal A		CBP (*n* = 125)		CBP vs. luminal A	
							OR (95% CI)^a^	*P* value^a^			OR (95% CI)^a^	*P* value^a^
TDLU count/100 mm^2^												
Median (IQR)	18.3	(9–35)	37	(18.0–65.0)	42	(20.0–75.0)			46	(20–75)		
Tertile 1	168	33.3	87	34.3	81	32.4	Reference		41	32.8	Reference	
Tertile 2	168	33.3	87	34.3	81	32.4	0.83 (0.47–1.49)	0.14	40	32.0	0.83 (0.41–1.67)	0.18
Tertile 3	168	33.3	80	31.5	88	35.0	1.44 (0.77–2.70)	0.09	44	35.2	1.23 (0.71–3.28)	0.11
OR_trend_/*P*_trend_							1.18 (0.86– 1.62)	0.31			1.24 (0.84– 1.82)	0.28
Maximum TDLU span (μm)												
Median (IQR)	775	(589.5–1023.0)	764.5	(600.0–992.0)	803	(588.0–1043.0)			809	(624.0–1100.0)		
Tertile 1	168	33.3	83	32.7	85	34.0	Reference		39	31.2	Reference	
Tertile 2	169	33.5	91	35.8	78	31.2	0.69 (0.39–1.22)	0.05	38	30.4	0.78 (0.39–1.57)	0.12
Tertile 3	167	33.1	80	31.5	87	34.8	1.25 (0.67–2.30)	0.13	48	38.4	1.53 (0.73–3.18)	0.08
OR_trend_/*P*_trend_							1.12 (0.82– 1.52)	0.48			1.26 (0.87– 1.82)	0.22
Maximum category of acini count/TDLU												
Median (IQR)	3	(2.0–4.0)	3	(2.0–4.0)	3	(2.0–5.0)			3	(2.0–5.0)		
Tertile 1	176	34.9	97	38.2	79	31.6	Reference		39	31.2	Reference	
Tertile 2	211	41.9	103	40.6	108	43.2	1.57 (0.89–2.77)	0.70	47	37.6	1.24 (0.62–2.47)	0.27
Tertile 3	117	23.2	54	21.3	63	25.2	2.06 (1.03–4.11)	0.09	39	31.2	2.89 (1.27–6.55)	0.006
OR_trend_/*P*_trend_							1.42 (1.01– 2.01)	0.045			1.69 (1.12– 2.55)	0.012

*Abbreviations: CBP* Core basal phenotype, *TDLU* Terminal duct lobular unit, *TN* Triple-negative

^a^ORs, 95% CIs, and *P* values were obtained from logistic regression analyses comparing cases with TN/CBP tumors or CBP tumors only with cases with luminal A tumors, adjusted for age (5-year intervals), body mass index (<23, 23–24.9, 25–29.9, or 30+ kg/m^2^), parity (nulliparous, one child, or more than one child) and grade (well, moderately, or poorly differentiated)

**Table 3:** Association of terminal duct lobular unit involution with breast tumor subtypes, by age group

	Age <50 years										Age ≥50 years									
	Luminal A (*n* = 124)		TN/CBP (*n* = 128)		TN/CBP vs. luminal A		CBP (*n* = 65)		CBP vs. Luminal A		Luminal A (*n* = 130)		TN/CBP (*n* = 122)		TN/CBP vs. luminal A		CBP (*n* = 60)		CBP vs. luminal A	
	Number	%	Number	%	OR (95% CI)^a^	*P* value^a^	Number	%	OR (95% CI)^a^	*P* value^a^	Number	%	Number	%	OR (95% CI)^a^	*P* value^a^	Number	%	OR (95% CI)^a^	*P* value^a^
TDLU count/100 mm^2^																				
Median (IQR)	29.7	(17.4–40.2)	30.4	(16.9–48.4)			30.8	(19.0–46.8)			11.7	(5.66–18.1)	11.62	(5.81–21.7)			10.1	(4.2–20.4)		
Tertile 1	39	31.5	45	35.2	Reference		20	30.8	Reference		43	33.1	41	33.6	Reference		26	43.3	Reference	
Tertile 2	50	40.3	34	26.6	0.57 (0.25–1.28)	0.03	22	33.9	0.94 (0.34–2.58)	0.28	47	36.2	37	30.3	0.59 (0.26–1.35)	0.11	14	23.3	0.31 (0.11–0.84)	0.02
Tertile 3	35	28.2	49	38.3	1.58 (0.70–3.57)	0.04	23	35.4	2.29 (0.78–6.66)	0.06	40	30.8	44	36.1	1.11 (0.48–2.59)	0.32	20	33.3	0.77 (0.31–1.94)	0.44
OR_trend_/*P*_trend_					1.27 (0.85– 1.91)	0.25			1.52 (0.89– 2.61)	0.13					1.05 (0.69– 1.60)	0.81			0.86 (0.54– 1.38)	0.54
Maximum TDLU span (μm)																				
Median (IQR)	904.0	(705.5–1195.5)	944.0	(750–1161.5)			985.0	(796–1220)			664.0	(503–819)	638	(477–826)			645.0	(483.5–820)		
Tertile 1	48	38.7	36	28.1	Reference		17	26.2	Reference		44	33.9	40	32.8	Reference		19	31.7	Reference	
Tertile 2	35	28.2	49	38.3	2.12 (0.96–4.66)	0.13	23	35.4	2.03 (0.74–5.54)	0.43	42	32.3	42	34.4	0.75 (0.32–1.75)	0.39	22	36.7	0.87 (0.33–2.31)	0.77
Tertile 3	41	33.1	43	33.6	1.55 (0.70–3.45)	0.85	25	38.5	2.06 (0.75–5.67)	0.40	44	33.9	40	32.8	1.04 (0.44–2.43)	0.62	19	31.7	0.96 (0.36–2.54)	0.94
OR_trend_/*P*_trend_					1.25 (0.84– 1.85)	0.28			1.43 (0.87– 2.36)	0.16					1.03 (0.67– 1.58)	0.89			0.99 (0.61– 1.60)	0.95
Maximum category of acini count/TDLU^b^																				
Median (IQR)	4.0	(3–5)	4.0	(3–5)			4.0	(3–5)			2.0	(2.3)	2	(2–4)			2.0	(1–3)		
Tertile 1	46	37.1	39	30.5	Reference		17	26.2	Reference		76	58.5	63	51.6	Reference		32	53.3	Reference	
Tertile 2	34	27.4	38	29.7	1.61 (0.71–3.68)	0.62	17	26.2	1.63 (0.55–4.86)	0.59	54	41.5	59	48.4	1.40 (0.71– 2.75)^b^	0.33^b^	28	46.7	1.25 (0.58– 2.69)^b^	0.57^b^
Tertile 3	44	35.5	51	39.8	1.82 (0.82–4.00)	0.30	31	47.7	4.39 (1.45–13.23)	0.009										
O_Rtrend_/*P*_trend_					1.34 (0.90– 1.99)	0.14			2.11 (1.22– 3.67)	0.008										

*Abbreviations: CBP* Core basal phenotype, *TDLU* Terminal duct lobular unit, *TN* Triple-negative

^a^ORs, 95% CIs, and *P* values were obtained from logistic regression analyses comparing cases with TN/CBP tumors or CBP tumors only with cases with luminal A tumors, adjusted for age (5–year intervals), body mass index (<23, 23–24.9, 25–29.9, or 30+ kg/m^2^), parity (nulliparous, one child, or more than one child), and tumor grade (well, moderately, or poorly differentiated)

^b^Number of acini was analyzed as the binary variable (greater than or equal to the median vs. below the median). Tertile categorization was not used, owing to the uneven distribution in acini count/TDLU among older women

## Discussion

In this study, we demonstrated that some measures of TDLU involution are reduced in breast parenchyma associated with TN/CBP as compared with luminal A cancers among Chinese women, particularly those younger than age 50 years, which is generally similar to our previously reported finding among Polish women [14]. Our finding adds to the accumulating evidence that the etiology and pathogenesis of these breast tumor subtypes differ.

Age-related TDLU involution has been associated with a number of breast cancer risk factors, including age, BMI, parity, family history of breast cancer, menopausal hormone therapy, and MD, in healthy women or women with benign breast disease [6–8]. In this study of Chinese patients with breast cancer, we observed associations of age, parity, and breastfeeding with TDLU involution measures similar to those shown in previous studies. Together, these findings suggest that measures of TDLU involution may reflect lifetime exposures to breast cancer risk factors and therefore may provide etiologic clues to breast tumor subtypes. For example, parity is known to protect ER^+^ breast cancer but may increase the risk of TN/CBP cancer [13, 16]. Parity has consistently been shown to be associated with less TDLU involution in a number of studies [4, 6, 14] and may explain the increase in risk for CBP breast cancer, whereas the parity-related protection for ER^+^ cancer may be mediated through a different mechanism that is independent of TDLU involution. Similarly, breastfeeding was associated with more TDLU involution among premenopausal women but with less involution among postmenopausal women [6]. Studies of African Americans showed that long-term breastfeeding had a stronger protective effect for TN than for luminal cases [16], suggesting that TDLU involution may partially mediate the protective effect of breastfeeding, particularly among younger women. Previous studies also associated having a positive family history of breast cancer with less TDLU involution [6], consistent with the observation that family history is more prevalent in cases with CBP tumors. These results, together with previously reported subtype differences in molecular mechanisms related to breast cancer risk factors such as parity [17], suggest that breast cancer risk factors may influence molecular pathways differently in different subtypes with distinct biological characteristics of at-risk tissue, which is manifested as TDLU involution.

According to the “breast tissue aging” concept proposed by Pike et al. [18], breast epithelium and stroma should be influenced by similar factors, such as age and parity [19]. Consistent with this notion, MD and TDLU involution have been shown to be correlated: Women with higher MD are likely to show lesser degrees of TDLU involution [7, 20]. Although correlated, reduced TDLU involution and elevated MD have also been shown to be independently associated with increased breast cancer risk [8]. However, MD has not consistently been associated with specific breast cancer subtypes [21]. Our finding of an involution-subtype association suggests that TDLU involution, which reflects changes that are specific in breast epithelium, may better capture the subtype-specific carcinogenic process than MD. Of note, the histologic feature that is more strongly correlated with MD is TDLU span [7], whereas data from this study and the previous analysis of Polish women revealed that subtype was most closely linked to acini per TDLU. This is consistent with the histologic observation that acini and epithelial content may decline even when the boundaries of TDLUs remain largely unchanged. Among the three metrics of TDLU involution, TDLU span and acini count per TDLU are usually highly correlated, whereas they show only weak correlations with TDLU count [6, 7]. Together with the various associations of breast cancer risk factors with specific TDLU metrics [6], these observations suggest that different involution variables may reflect different biological mechanisms or stages of TDLU involution and that acini count per TDLU measure may be more relevant to capturing tumor subtype-specific pathology. Alternatively, the differences may also reflect variations in tissue sampling. Nevertheless, the associations with tumor subtype for all three TDLU metrics showed the same directions in both overall and stratified analyses, suggesting that the TDLU involution-subtype association is not specific to certain involution measurements.

Studying interrelationships between histologic and radiologic measures of breast tissue composition in Chinese women is an area requiring further exploration. In addition, we found that the association of acini per TDLU with tumor subtype was stronger when using maximal rather than median acinar value as the summary measurement for each subject, despite the high correlation between the two metrics (Spearman’s *r* ^2^ = 0.83), suggesting that the maximal number of acini count per TDLU may provide important information in terms of subtype associations.

Our study is the first study to describe features of TDLU involution among Asian women using three standardized quantitative measures including TDLU count per unit area, which was not previously assessed in the Polish study. In Polish cases [14], we restricted the analysis to younger women (aged <55 years) and women with observed TDLUs. In the present study, we extended the evaluation to a broader age range because the intact TDLU structure was persistent even among very old women in this Chinese population. The magnitude of association between acini count and CBP tumor among younger (aged <50 years) Chinese cases (OR_T3 vs. T1_ 4.39) was similar to what was observed among Polish cases (OR_T3 vs. T1_ 4.44). Given the considerable differences in the prevalence of breast cancer risk factors such as BMI and MD in Chinese and Western women, future research is needed to investigate whether the natural history of TDLU involution is different in diverse populations.

Several limitations of our study should be noted. First, our study is limited by the relatively small sample size, especially in stratified analyses by age and in the analysis restricted to CBP tumors. Second, we defined the luminal A subtype based on ER, PR, HER2, and basal markers only, and therefore a number of high-grade luminal tumors were included. Future studies with more accurate tumor subtype information are needed to better quantify the association. Nevertheless, the replication of a TDLU involution-subtype association in an independent population highlights the importance of studying TDLU involution as a possible intermediate endpoint in breast cancer carcinogenesis. Because of the cross-sectional design of our study, we were not able to evaluate whether concurrent TDLU involution features associated with tumor subtype preceded tumor development or occurred later during cancer progression. However, our previous study among cancer cases indicated high correlations between TDLU metrics (e.g., acini count and diameter) within an individual, regardless of tumor proximity (proximal vs. distant normal to tumor) [14]. In addition, researchers in a study of women with benign breast disease also reported a high level of agreement in involution measures across multiple biopsy specimens within a woman [2]. These observations suggest that the observed involution-subtype association is less likely to be driven by tumor progression. Nevertheless, more detailed studies with prospective designs are needed to definitively assess the temporal and spatial relationships between TDLU involution and tumor subtypes. Finally, information on some established breast cancer risk factors that may potentially influence both TDLU involution and tumor subtypes, such as menopausal hormone therapy, was not collected in our study. However, given the low menopausal hormone therapy prevalence among Chinese women [22] and low population-attributable risk of breast cancer due to menopausal hormone therapy in Asian women in general [23, 24], it is unlikely that the lack of information on menopausal hormone therapy had a significant impact on the association between TDLU involution and tumor subtype in postmenopausal women.

In summary, we have confirmed the previously observed association between greater acini count per TDLU (reflecting reduced TDLU involution) and the TN subtype in an independent Asian population. Our results further support the different etiology and pathogenesis of breast cancer subtypes and highlight the importance of investigating TDLU involution as a possible intermediate endpoint in breast cancer carcinogenesis among diverse populations. Further, future efforts to combine TDLU involution metrics with follow-up data may lead to important translational findings on local recurrence and treatment efficacy.

## Conclusions

In this study of Chinese women, a group with typically lower breast cancer incidence rates and denser breasts than Western women, we showed again that TN/CBP cases had a significantly higher acini count per TDLU than luminal cases. Our findings strengthen and extend the previous observation that involution is associated with breast cancer subtype, which may have implications for understanding the pathogenesis of breast cancer. Future investigations in large studies and diverse populations are needed to further explore ethnic differences in TDLU involution in relation to etiologic factors and tumor characteristics.

## Additional file

Additional file 1: Table S1. Characteristics of Chinese breast cancer cases by tumor subtype. **Table S2.** The association of TDLU involution (median TDLU span and median acini count/TDLU) with breast tumor subtypes, overall and by age group. (XLS 64 kb)

## Abbreviations

BMI: Body mass index
CBP: Core basal phenotype
CHCAMS: Cancer Hospital, Chinese Academy of Medical Sciences
CK: Cytokeratin
EGFR: Epidermal growth factor receptor
ER: Estrogen receptor
FISH: Fluorescence in situ hybridization
H&E: Hematoxylin and eosin
HER2: Human epidermal growth factor receptor 2
IHC: Immunohistochemistry
MD: Mammographic density
PR: Progesterone receptor
TDLU: Terminal duct lobular unit
TN: Triple-negative
